# Supercritical Carbon Dioxide-Based Processes in Photocatalytic Applications

**DOI:** 10.3390/molecules26092640

**Published:** 2021-04-30

**Authors:** Paola Franco, Olga Sacco, Vincenzo Vaiano, Iolanda De Marco

**Affiliations:** 1 Department of Industrial Engineering, University of Salerno, Via Giovanni Paolo II, 132, 84084 Fisciano, Italy; pfranco@unisa.it (P.F.); vvaiano@unisa.it (V.V.); 2 Department of Chemistry and Biology “A. Zambelli”, University of Salerno, Via Giovanni Paolo II, 132, 84084 Fisciano, Italy; osacco@unisa.it; 3 Research Centre for Biomaterials BIONAM, University of Salerno, Via Giovanni Paolo II, 132, 84084 Fisciano, Italy

**Keywords:** supercritical drying, supercritical antisolvent, supercritical impregnation, heterogeneous photocatalysis, air pollution, wastewater treatment

## Abstract

Conventional methods generally used to synthesize heterogeneous photocatalysts have some drawbacks, mainly the difficult control/preservation of catalysts’ morphology, size or structure, which strongly affect the photocatalytic activity. Supercritical carbon dioxide (scCO_2_)-assisted techniques have recently been shown to be a promising approach to overcome these limitations, which are still a challenge. In addition, compared to traditional methods, these innovative techniques permit the synthesis of high-performance photocatalysts by reducing the use of toxic and polluting solvents and, consequently, the environmental impact of long-term catalyst preparation. Specifically, the versatility of scCO_2_ allows to prepare catalysts with different structures (e.g., nanoparticles or metal-loaded supports) by several supercritical processes for the photocatalytic degradation of various compounds. This is the first updated review on the use of scCO_2_-assisted techniques for photocatalytic applications. We hope this review provides useful information on different approaches and future perspectives.

## 1. Introduction

Techniques based on the use of supercritical fluids have been used for more than 50 years because of their physicochemical properties, which are intermediate between a gas and a liquid and are easily adjustable with changes in temperature and pressure. Among the different fluids that are used at supercritical conditions; i.e., at pressures and temperatures higher than the critical values, carbon dioxide is the most employed one, because of its mild critical conditions (*T*_c_ = 31.1 °C, *P*_c_ = 7.38 MPa), its recyclability, cheapness, and low toxicity. Moreover, considering that carbon dioxide is a gas at room temperature and pressure, solvent-free products can be obtained after the depressurization at ambient pressure. Techniques based on the use of supercritical carbon dioxide (scCO_2_) have been used as an alternative to different traditional processes such as extraction, micronization, impregnation, membrane and aerogel formation, electrospinning, electrospray and so on [1,2,3,4,5]. Considering the variety of techniques used, even the fields of application are among the most diverse, from pharmaceutical/biomedical [6,7,8] to food packaging [9,10], and from dye processing [11,12] to the catalytic field [13,14].

Focusing on the catalytic field, it is possible to note that from same perspective of using environmentally friendly processes, the interest of the scientific community in heterogeneous photocatalysis (HP) has grown considerably. HP is an advanced oxidation process in which the inexhaustibly abundant, clean, and safe solar energy is converted and stored through reactions involving electron transfer. HP is a versatile, efficient, relatively cheap, and environmentally benign technology for decomposing various biological, organic, and inorganic pollutants present in low concentrations within the soil, water, and air [15,16]. Its main advantages are: (1) it is a good substitute for conventional energy-intensive treatments, considering that it uses renewable sources of energy and solar energy; (2) differently from conventional treatments, which transfer pollutants from a medium to another, harmless products are formed due to the photocatalytic reaction; (3) it can be applied to aqueous, gaseous and soil treatments; (4) its reaction conditions are mild, the reaction time is modest, and a small amount of chemical reagents is required [17]. 

The materials generally employed in photocatalysis are semiconductors with a typical band structure, roughly characterized by a valence band, which is the highest energy band in which all electronic levels are occupied by electrons and a conduction band, which is the lowest energy band not occupied by electrons [18,19]. The energy intervals between the electronically populated valence band and the vacant conduction band, called band gaps, constitute forbidden energy intervals, which electrons cannot assume [20]. The semi-conductive photocatalysts generally used are titanium dioxide or titania (TiO_2_) [21,22,23], ZnO [24,25], WO_3_ [26,27], Fe_2_O_3_ [28,29], CeO_2_ [30,31], MoO_3_ [32,33], ZrO_2_ [34,35], SnO_2_ [36,37], CdS [38,39], and ZnS [40,41]. Up to now, semiconductor-based photocatalysts in various forms have been proposed, mainly as nanopowders [42,43,44,45,46,47,48] or aerogels [49,50,51,52,53,54], due to the high specific surface area of these materials, resulting in a very high contact between the semiconductor and the fluid to be treated and, consequently, the photocatalytic performance could be enhanced.

In recent years, the advantages of using processes based on the use of scCO_2_ have been exploited to obtain higher-performing photocatalysts for water and air purification. The environmental advantage resulting from the coupling of processes with a low environmental impact if compared to classical processes is undeniable. Indeed, several papers [55,56,57] have highlighted the potential of exploiting scCO_2_ in terms of catalytic and environmental performances mainly related to the replacement of hazardous, polluting and toxic organic solvents or reaction media.

To the best of our knowledge, no reviews have been published on the use of supercritical carbon dioxide-based techniques for obtaining photocatalysts. Therefore, the present review is organized by classifying the published papers according to the supercritical carbon dioxide-based process used to produce the photocatalyst. Indeed, different approaches have been investigated, such as supercritical deposition, supercritical antisolvent process to obtain nanoparticles, sol–gel reaction, supercritical drying, and others.

## 2. Supercritical Deposition of Nano-Scale Metal-Organic Precursors

**Figure 1:**
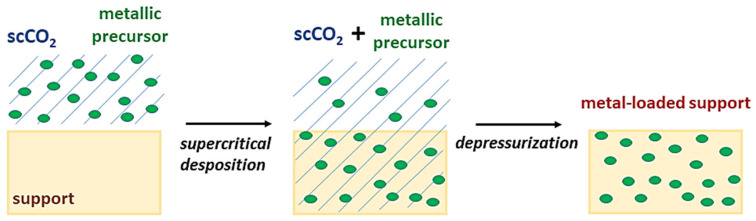
Sketch of supercritical deposition stages.

Nanoparticles, nanorods, aerogels or thin films have been frequently used in photocatalysis because of the high surface-to-volume ratio of nanostructured materials. Metals and metal oxides can be dispersed as nanoparticles on both the external and the internal surface of support materials. Supercritical deposition has been attracting interest because of the peculiar properties of scCO_2_, such as solvent power adjustable with small variations in pressure and temperature, absence of liquid waste and solvent residue on the substrate, fast rates of deposition because of high mass transfer rates at supercritical conditions [58,59]. According to Watkins and McCarthy [60], it is possible to consider three subsequent steps (Figure 1):

1. The metal precursor is dissolved in scco_2_;
2. The metal precursor is adsorbed from the fluid phase to the support material or reacts with the surface of the support;
3. The adsorbed metal precursor is converted to its metal or metal oxide form.

Watkins and McCarthy [60] also highlighted the numerous advantages in the preparation of polymer/metal nanocomposites using this supercritical approach. Specifically, a key aspect is the ability of the scCO_2_ to swell the polymeric substrates that, combined with the high scCO_2_ diffusivity, leads to high penetration of organic/organometallic reagents solubilized in scCO_2_ into the polymer matrix. The degree of polymer swelling and diffusion/transport properties can be modulated by varying the temperature and pressure, which strongly influence the density and the solvent power of scCO_2_. However, the polymer constituting the support as well as the reaction product has to be insoluble/poorly-soluble in the supercritical fluid. In addition, the use of scCO_2_ offers an outstanding control of the composites’ morphology; e.g., it can allow the preservation of the porous structure of matrices, avoiding their collapse, or the spherical nanoparticle shape, with reduced sintering phenomena. Aiming at the photocatalytic degradation of methylene blue dye, Horibe et al. [61] loaded TiO_2_ nanoparticles on/in nanostructured-carbon walls by the supercritical deposition of titanium tetraisopropoxide (TTIP), which was selected as the organometallic precursor. Specifically, two reactions were involved in the formation of TiO_2_: firstly, the decomposition of the precursor TTIP, followed by the dehydration of the intermediate obtained by the first stage. A better nucleation and growth of the nanoparticles on the carbon nanowalls surface occurred at 180 °C with respect to 100 °C, leading to the attainment of a support in which the TiO_2_ nanoparticles were homogeneously dispersed. 

Similarly, Liu et al. [62] and Kashiwaya et al. [63] prepared photocatalysts by supercritical decomposition, to be applied for the photocatalytic removal of two dyes, methylene blue, and methyl orange. In particular, Liu et al. [62] proposed a novel method of “supercritical deposition assisted by liquid-crystal template” to prepare nanocomposites consisting of mesoporous silica SBA-15 coated with TiO_2_. Compared to the results obtained by coating the TiO_2_ on the surface of non-porous SBA-15 using a similar approach without template, mesoporous TiO_2_ coated SBA-15 showed a more uniform deposition and an improved degradation efficiency for azo dyes as well as phenol. In the study of Kashiwaya et al. [63], the scCO_2_ allowed to effectively impregnate nitrate nickel oxide (NiO) nanoparticles, starting from hexahydrate (Ni(NO_3_)_2_ 6H_2_O) as the organometallic precursor, on the (101)-facet of the oriented TiO_2_ nanoparticles. In this way, heterostructure NiO/(101)-anatase-TiO_2_ nanoparticles were synthesized, characterized by good dispersion of NiO. A NiO loading equal to 0.25 wt% into the composites exhibited the best photocatalytic activity, which was much higher than that of the pure TiO_2_ photocatalyst. Moreover, it was proved that no changes occurred in the textural and morphological properties of the employed anatase-TiO_2_ nanoparticles after the supercritical deposition of NiO loadings.

Aiming at the production of high-performance nanocomposites, Sun et al. [64] dispersed the coupling system ZnO/NiO, in the form of nanoparticles, on ordered mesoporous alumina (OMA). The OMA supported-ZnO/NiO showed a higher photocatalytic activity for Congo Red decolorization under simulated sunlight irradiation not only with respect to ZnO/OMA and NiO/OMA composites, but also compared to OMA/ZnO/NiO composites synthesized by the conventional incipient wetness impregnation. These results revealed the potential of the supercritical deposition route to achieve an excellent dispersion of the metallic compounds on the selected support.

In Table 1, a summary of the studies focused on the production of composites by the supercritical deposition for photocatalytic applications is shown.

## 3. Supercritical Antisolvent Precipitation of Photocatalytic Nanoparticles

Up to now, the Supercritical AntiSolvent (SAS) precipitation was widely used to produce nanoparticles of different materials, including metal-based compounds to produce catalysts [66,67,68,69] and photocatalysts [42,43,44,45,46] with very high activity and selectivity performance for various reactions.

The SAS process is based on some fundamental prerequisites. Specifically, the scCO_2_ plays the role of the antisolvent with respect to the solute to be precipitated, whereas it has to be completely miscible with the selected liquid solvent. On the other hand, the solute to be nanonized has to be soluble in the liquid solvent and insoluble in the binary mixture solvent/antisolvent formed in the precipitation chamber during the process. In addition to the fast diffusion of scCO_2_ into the liquid solvent, the precipitation is thus caused by the supersaturation of the solute. Organometallic precursors of the desired oxides are generally dissolved in liquid solvents to prepare photocatalysts. Briefly, the liquid solution consisting in the solvent and the precursor is injected into the precipitator filled with the scCO_2_ at the selected temperature and pressure, leading to the attainment of nanoparticles. Then, the as-prepared precursor particles have to be usually calcined at high temperatures to produce the final oxide photocatalyst. Isopropanol, dimethylsulfoxide (DMSO), ethanol and methanol are usually selected as the proper solvents for the precipitation of photocatalyst precursors [42,43,44,45,46].

**Figure 2:**
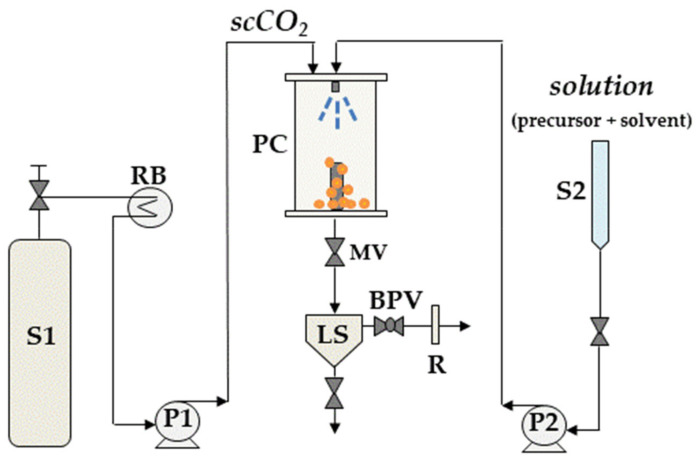
A schematic representation of a generic SAS process. P1, P2: pumps; S1: CO_2_ supply; S2: liquid solution supply; RB: refrigerating bath; PC: precipitation chamber; LS: liquid separator; MV: micrometric valve; BPV: back-pressure valve; R: rotameter.

A typical sketch of a generic SAS process is shown in Figure 2.

To date, the SAS technique was mainly employed to produce TiO_2_ nanoparticles, starting from titanium tetraisopropoxide as precursor [42,43,44]. Da Silva et al. [43] highlighted that SAS precipitation allows to produce photocatalyst nanoparticles with very high specific surface areas, up to 515 m^2^/g in the specific case of TiO_2_ nanoparticles prepared by the authors. Moreover, there was a correlation between the photocatalytic activity of TiO_2_ and its phase composition as well as because of synergism phenomena between different titania phases. In particular, the anatase-rutile ratio strongly affected the photodegradation of methylene blue and methyl orange: for this purpose, the sample composed of 7% rutile and 93% anatase emerged as the best one. SAS TiO_2_ samples were also superior compare to the commercial photocatalyst known as P25, which is a widely used TiO_2_ consisting of anatase and rutile phases. Similarly, Marin et al. [44] prepared TiO_2_ with a tailored anatase/rutile composition via SAS process, but the SAS-precipitated TiO_2_ was found to have a comparable activity with commercial TiO_2_ one for the photocatalytic splitting of water for the hydrogen production.

In another study [42], SAS-prepared nanocrystalline TiO_2_ revealed to be effective also in the photodegradation of a dye, namely Reactive Red 180 (RR 180), in water solutions, mainly due to the small particle size, the high surface area (surface area of 63.2 m^2^/g) and weak agglomeration of the produced powders.

However, generally speaking, the use of TiO_2_-based photocatalysts is limited by some critical issues, such as the deactivation caused by ions scavengers present in the solution to be treated and low degradation kinetics in solutions with high concentrations of the contaminant to be removed [70]. The superior photocatalytic activity of ZnO compared to TiO_2_ is well documented [24,71,72].

Recently, undoped ZnO and ZnO doped with Europium (Eu) were also synthesized via the SAS process [45,46]. Specifically, in the studies of Franco et al. [45,46], undoped ZnO nanoparticles precipitated by SAS were found to be effective in the photocatalytic degradation of both Crystal Violet dye and Eriochrome Black-T Azo dye, even to a greater extent than a TiO_2_ photocatalyst, in addition to ZnO prepared using traditional methods. This outcome was assured by the regular nanometric shape, the low particle size and a high exposed surface area of SAS-prepared ZnO. The authors also highlighted the importance of carefully selecting the proper conditions for the thermal treatment of the SAS-prepared precursor to preserve the nanoparticle morphology and size even in the final metal-oxide photocatalyst. Specifically, a calcination temperature equal to 500 °C (temperature range investigated: 300–600 °C) in addition to a slow heating rate equal 2 °C × min^−1^, for 2 h in air, was revealed to be the best choice to avoid sintering phenomena. In addition, spectroscopic studies proved that organic impurities, which are present in ZnO obtained from unprocessed ZnAc, completely disappeared after the supercritical processing in the SAS powders. This outcome could further explain the superior photocatalytic performance of SAS-prepared powders. 

Aiming to increase the photocatalytic performance of SAS ZnO, a novel simultaneous precipitation of zinc acetate (ZnAc) and europium acetate (EuAc) via SAS process was also proposed by the authors to obtain Eu-doped ZnO [46]. Indeed, SAS Eu-doped ZnO ensured the highest photocatalytic efficiency in terms of discoloration and mineralization of the Eriochrome Black-T Azo dye. As a future prospect, it could be interesting to investigate the simultaneous precipitation of multiple metal precursors to produce doped-photocatalysts.

**Figure 3:**
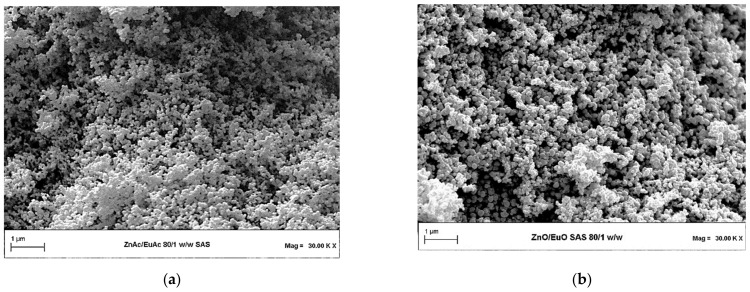
FESEM images of SAS nanoparticles of (**a**) Eu-ZnAc before thermal calcination and (**b**) Eu-ZnO after calcination step.

Two illustrative images obtained by Field Emission Scanning Electron Microscopy (FESEM) are shown in Figure 3a,b, which show SAS Eu-ZnAc nanoparticles and SAS Eu-doped ZnO photocatalyst, respectively, obtained after the calcination of SAS Eu-ZnAc.

Table 2 shows a summary of the studies focused on the application of the SAS precipitation to prepare nanoparticulate photocatalysts.

## 4. Sol–Gel Reactions

Sol–gel reactions in scCO_2_ have been frequently used for the synthesis of metal/silicon oxides with different geometries on a nanometer scale [73]. In sol−gel processes, the sol is a colloidal suspension of nanometer-sized solid particles in a liquid phase; the gel is formed when the particles bond together forming a three-dimensional network. The use of scCO_2_ as the reaction media involves many advantages and has been used starting from 1996, when Tadros et al. [74], for the first time, synthesized anatase TiO_2_ microparticles (mean diameter 0.1–2.0 μm) using TTIP, an aqueous solution of a surfactant and scCO_2_. The pressure was fixed at 9.9 MPa, the temperature at 50 °C and the reaction time at 4 h. Subsequently, Reverchon et al. [75] hydrolyzed TTIP in scCO_2_ to obtain Ti(OH)_4_ nanoparticles with a mean particle size in the range 90–130 nm. The operating pressure ranged from 8 to 14 MPa, whereas the operating temperature ranged from 40 to 60 °C.

Jensen et al. [76] produced nanosized metal oxides through a supercritical seed enhanced crystallization, a modified sol–gel process in which scCO_2_ is the solvent and different seeding materials can be used. The process was developed at a pressure of 10 MPa, a temperature of 100 °C and considering a process time equal to 4 h. Depending on the seeding material, anatase TiO_2_ powder with a mean crystal size in the range 6.2–9.3 nm were obtained. The nanoparticles were used in a subsequent paper [77] to prepare titania nanocrystalline films through a modified sol–gel method. The photocatalytic activity of the films was determined under UV irradiation using stearic acid as a model compound.

Camarillo et al. [78] obtained titania nanofibers (undoped and doped with Cu) through a synthesis in scCO_2_ that was used for the photocatalytic reduction of carbon dioxide with water vapor. The nanofibers were produced using TTIP as precursor of Ti, Copper (II) acetylacetonate as precursor of Cu, and acetic acid as polymerizing agent. Carbon dioxide is pumped into the reactor that contained the previous materials up to the operating pressure (22–24 MPa) and temperature (40–80 °C). During the first three hours of the reaction the mixture is kept under stirring and, subsequently, the reaction is aged for 24 h under the same pressure and temperature conditions in the absence of stirring. The photocatalytic reduction of CO_2_ was conducted using the catalyst, obtaining methane and carbon monoxide as the only reaction products. 

## 5. Sol–Gel Reactions and Supercritical Drying to Obtain Porous Structures

**Figure 4:**
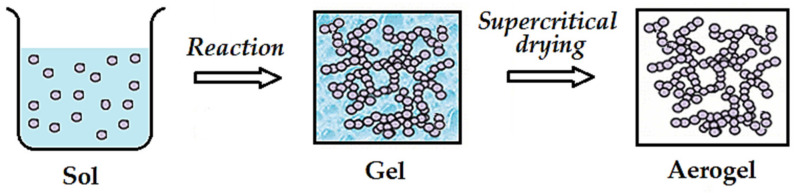
A sketch of sol–gel and supercritical drying stages.

In some papers, after the sol–gel reaction, a supercritical drying is assessed (Figure 4), obtaining aerogels instead of xerogels since the last ones can suffer from severe shrinkage and loss of microstructure when dried by conventional evaporation.

Up to now, TiO_2_-based photocatalysts have been generally prepared by supercritical drying, mostly in form of aerogels [51,79,80,81,82,83]; however, other novel structures have also been proposed; e.g., nanowires [84], thin films [85], pillared clays [86] or beads [87].

Mumin et al. [84] synthesized anatase TiO_2_ nanowires through a sol–gel process in scCO_2_; the precursor of the oxide was TTIP, which was dissolved in acetic acid and put in contact with scCO_2_ at 41.4 MPa and 60 °C for 24 h under stirring and 5 days of aging (to complete the reaction). Then, a supercritical drying step was performed. The photocatalyst was, then, prepared by functionalizing TiO_2_ nanowires and linking covalently semiconductor quantum dots to the titania surface. The photocatalytic activities of the prepared catalysts have been evaluated under ultraviolet and visible light solar irradiation for the photodegradation of methylene blue (MB). In particular, among various proposed and tested samples, the best performance in terms of photodegradation of MB dye (approximately equal to 88%) were exhibited by the nanocomposites consisting in core-shell CdS-ZnS quantum dots linked with TiO_2_ nanowires. A remarkable aspect is the stability of this nanocomposite system over time; indeed, after three cycling tests, the loss in terms of photocatalytic activity was significantly reduced up to a value of about 10%. On the other hand, for the MB photodecomposition, Li et al. [49] proposed TiO_2_–WO_3_-Fe^3+^ aerogels prepared using milder conditions in the supercritical drying phase in terms of pressure, temperature and process time (as observable in Table 3), compared to those employed in the study of Mumin et al. [84]. This aspect can be advantageous from both an economic and production time point of view for the long-term industrial preparation of photocatalysts.

Aiming at improving the photocatalytic activity of catalysts, especially those based on TiO_2_, different approaches have been attempted, including the dispersion of an active species onto the support surface (in general, a noble metal such as Au [87] and Pt [88]), as well as the photocatalyst doping [89,90]. In particular, the doping of TiO_2_ is a promising route to overcome the main issues associated to its use in photocatalysis, including the wide band gap (3–3.2 eV) that requires ultraviolet irradiation for its photocatalytic activation, resulting in a very low energy efficiency under solar light, whose use is still today the main challenge and desired goal. Specifically, TiO_2_ was doped with both non-metal elements, such as nitrogen [89,91], and transition metal ions, such as Fe(III) [90], involving a final step of supercritical drying to remove the liquid solvent employed, while preserving the native porous structure of the aerogels. In addition to the doping, the use of bimetallic materials is also a valid route to improve the photocatalyst features and performance, as proven in the study of Lucky and Charpentier [91]. Specifically, the authors prepared N-doped ZrO_2_/TiO_2_ nanomaterials, observing that a small and proper amount of nitrogen and zirconia led to an increase in the specific surface area and inhibited the crystals’ growth, thus assuring a low degree of crystallinity in the samples. As a result, all the synthesized N-doped ZrO_2_/TiO_2_ samples exhibited a higher activity in photodegradation of methylene blue compared to commercial P25 TiO_2_.

Recently, a biopolymer-templating methodology has also been proposed in some papers [31,87]. Natural alginate is the most selected material as a templating agent for the preparation of metal oxide structures, since it allows to achieve very small particle size and/or very high specific surface area. This result is due to the fact that the transition metals are contained in the alginate once dried in the presence of supercritical CO_2_.

To date, TiO_2_–SiO_2_ composites involving supercritical drying and/or sol–gel reactions in scCO_2_ have also been widely proposed for various photocatalytic applications, including water splitting, degradation of benzene, phenol and methanol, oxidation of volatile organic compounds (VOCs) or trichloroethylene [50,83,86,92,93,94]. In the studies of Cao [93,94], mesoporous titania–silica aerogels with an open-pore structure readily accessible to the reactant molecules have been obtained using scCO_2_; consequently, a great contact between the reactants and TiO_2_–SiO_2_ led to a high conversion. Specifically, the nanometric crystals of anatase TiO_2_ (i.e., Ti–O–Ti sites) were well-dispersed and well-anchored within/to the amorphous aerogel network (i.e., Si–O–Ti and Si–O–Si bonds). Furthermore, under similar reaction conditions, catalysts based on commercial TiO_2_ exhibited lower activity performance than titania–silica aerogels by supercritical ethanol drying.

The studies focused on the application of the sol–gel reaction and the supercritical drying in the photocatalytic field are summarized in Table 3.

## 6. Other Applications of Supercritical CO_2_ in Photocatalysis: From Supercritical Foaming to scCO_2_ as a Promising Reaction Medium

Supercritical CO_2_ has also been exploited in the photocatalytic field for other multiple purposes.

For example, before the sol–gel reaction, a supercritical pre-treatment has been conducted in some cases, aiming at the production of composite systems such as TiO_2_-coated carbon surface [98,99] or TiO_2_-coated zeolite surface [100]. In particular, this preliminary pretreatment consists in the supercritical impregnation of a plugging agent (such as paraffin [98,100]) into porous carbon or zeolite (i.e., the support for TiO_2_) to form sealed substrates. On the other hand, post-treatments under supercritical conditions can also be performed on photocatalysts. For example, Wang et al. [101] prepared mesostructured TiO_2_ thin films supported on silicon substrates by spin coating. The supercritical post-treatment of the thin films led to a remarkable improvement in the thermal stability of the mesoporous coatings without affecting the optical transparency or the integrity of samples. Indeed, the unidirectional contraction of the films and the pores collapsing were avoided in the case of the photocatalysts post-treated by scCO_2_. TiO_2_ thin films after supercritical processing also exhibited a very high photoactivity in the degradation of waste organic compounds.

Up to now, supercritical CO_2_ has been widely used for the attainment of catalysts characterized by different structures/morphologies. In this context, Zhang et al. [102] exploited the scCO_2_ to modify Ruthenium-coordinated metal−organic frameworks (MOFs), promoting the formation of a highly mesoporous and microporous structure. The supercritical processing resulted in a high porosity into composites that facilitated the mass transport, an enhanced capability to adsorption visible-light and, consequently, a higher photocatalytic activity for the hydrogen production compared to those of pure MOF and MOF functionalized with Ru. Marković et al. [103] suggested a novel approach to prepare floating photocatalysts for the degradation of textile dyes, namely C.I. Acid Orange 7 (AO7) and C.I. Basic Yellow 28 (BY28). This route consisted in the foaming of poly(ε-caprolactone) (PCL) beads using scCO_2_, thus promoting the formation of a highly porous structure, subsequently loaded with TiO_2_ nanoparticles. A sustainable floatability of the photocatalyst based on PCL foams was observed for a long period of time.

Another interesting aspect is the use of scCO_2_ as a reaction medium [55], for example, in the thermal hydrolysis for the catalyst preparation [104,105,106,107]. In this context, Camarillo et al. [104] synthesized a Palladium-doped TiO_2_ photocatalyst via thermal hydrolysis assisted by supercritical CO_2_ as reaction medium, using two different TiO_2_ precursors (i.e., titanium tetraisopropoxide and diisopropoxititanium bis(acetylacetonate)) and palladium acetylacetonate. The main aim was to produce highly efficient photocatalysts for the reduction of CO_2_ to hydrocarbons, which will be used as more convenient fuels from both environmental and economic points of view, finally passing from a laboratory process to a commercial scale. The as-prepared photocatalysts showed improved properties, mainly higher surface area and absorbance of visible light, in addition to more efficient production of methane (up to 22 times), compared to those of commercial TiO_2_. Similar benefits in the photocatalytic reduction of CO_2_, employing TiO_2_-based catalysts prepared by scCO_2_, were also highlighted in other papers [47,106,107,108]. The exploit of scCO_2_ in the photoelectrochemical field has also been recently attempted [109,110]. Table 4 reports an overview of other scCO_2_ applications in photocatalysis.

An overview about the various applications of scCO_2_ in photocatalysis and the related advantages is shown in Table 5.

## 7. Conclusions

The versatility of the supercritical CO_2_ allows its application in photocatalytic fields for multiple purposes. Among them, the preparation of photocatalysts by scCO_2_-assisted processes was revealed to be a very promising route to assure high performance for different kinds of reactions. The supercritical techniques are useful to synthesize photocatalysts in various forms, from nanoparticles to aerogels, by preserving the native structure due to the absence of capillary pressure. Moreover, reduced use of toxic and polluting solvents, or even their total elimination, is involved during the supercritical processing, leading to a lower environmental impact involved in the long-term catalyst preparation. The attainment of solvent-free catalysts also avoids the presence of any poisons on their surface, which can cause catalyst deactivation. The photocatalysts prepared by supercritical processes generally exhibit a higher photoactivity compared to that of commercial catalysts. This improved performance is primarily due to a very high surface area of the materials, providing an excellent contact between the reactant molecules and the photocatalyst. 

For future perspectives, it is interesting to exploit the use of supercritical technologies in the preparation of doped photocatalysts in all forms, which is still limited to a few studies. Indeed, in addition to the deposition of noble metals onto the support surface, the doping of photocatalysts is a promising route to improve further the catalyst performance. It is also worth investigating the development of novel catalyst structures, for example, using supercritical foaming to produce floating photocatalysts. The main challenge in photocatalysis is still to produce photocatalysts that have to be very active not only under UV irradiation but especially under visible light, also considering an economic point of view. The use of the supercritical CO_2_ as a reaction medium is also an interesting aspect; it is advisable to attempt to extend it to a wide variety of reactions. For various reasons, the photocatalytic reduction of CO_2_ involving the use of scCO_2_ as a reaction medium is very industrially attractive. Indeed, it is possible to re-use the CO_2_ captured from the industrial emissions as a feedstock for the photochemical production of fuels or reaction intermediates in large-scale plants, combining both environmental and economic benefits.

## Figures and Tables

**Table 1:** Synthesis of photocatalysts by supercritical deposition. NPs = nanoparticles; MB = methylene blue; MO = methyl orange; RB = rhodamine B; TBOT= tetrabutyl orthotitanate; TTIP= titanium tetraisopropoxide; *P*_PC_ = preparation chamber pressure; *T*_PC_ = preparation chamber temperature; *P*_S_= deposition chamber pressure; *T*_S_= stage temperature; *t*= deposition period.

Metal Organic Precursor	Metal Oxide	Operating Conditions	Mean NPs Diameter	Photocatalytic Application	Results	Ref.
TTIP	TiO_2_	*P*_PC_ =11 MPa; *T*_PC_ =40 °C; *P*_S_ = 9 MPa; *T*_S_ = 100-180 °C; *t* = 30 min	2.7 nm	MB degradation	40% of MB removal after 240 min	[61]
Ti(OC(CH_3_)_3_)_4_	TiO_2_/SBA-15	*-*	2.6–15.3 nm	MB, MO, RB, and phenol degradation	The highest degradation efficiency with a TiO_2_ loading of 15 wt% and a calcination temperature of 400 °C	[62]
Ni(NO_3_)_2_ · 6H_2_O	NiO	*P*_PC_ =5 MPa; *P*_S_ = 22 MPa; *T*_S_ = 200 °C; *t* = 30 min	7 nm	MB or MO Degradation	0.25 wt% NiO–TiO_2_ showed the highest efficiency for both MB and MO degradation	[63]
Zn(NO_3_)_2_ ·6H_2_O/ Ni(NO_3_)_2_ ·6H_2_O	ZnO/NiO	*P*_S_ = 20 MPa; *T*_S_ = 50 °C; *t* = 4 h	21 nm	Congo Red Degradation	About 97% of dye removal after 240 min; superior performance of catalysts prepared by supercritical deposition than those of commercial TiO_2_ and of composites prepared via incipient wetness impregnation	[64]
TBOT	TiO_2_	*P*_S_ = 20 MPa	-	MB degradation	Complete MB discoloration after 80 min with scCO_2_- prepared catalyst; a superior efficiency than that of commercial TiO_2_	[65]

**Table 2:** Production of photocatalysts by SAS precipitation. CV = crystal violet; RR 180 = reactive red 180; EB = eriochrome black-T; MB = methylene blu; MO = methyl orange; titanium tetraisopropoxide (TTIP); *P* = operating pressure; *T* = operating temperature.

Metal Organic Precursor	Metal Oxide	Operating Conditions	Mean Crystallite Size	Photocatalytic Application	Band Gap	Results	Ref.
TTIP	TiO_2_	*P* = 10-15 MPa; *T* = 50-150 °C	13 nm	RR 180 degradation	-	98% of dye removal after 45 min with TiO_2_ prepared at 15 MPa/150 °C	[42]
TTIP	TiO_2_	*P* = 25 MPa; *T* = 60 °C	27 nm	MB and MO degradation	3.22 eV	The highest photodegradation with TiO_2_ consisted of 7 wt% of rutile and 93 wt% of anatase phase (98% discoloration in 90 min)	[43]
TTIP	TiO_2_	*P* = 12 MPa; *T* = 40 °C	13 nm	Water splitting	-	Conversion% equal to 75% in 3 h; selectivity around 85% in all the time range studied	[44]
Zn(CH_3_COO)_2_	ZnO	*P* = 15 MPa; *T* = 40 °C	18 nm	CV degradation	3.10 eV	500 °C as optimum calcination temperature to preserve the nanoparticle morphology; complete CV decolorization in 60 min	[45]
Eu(CH_3_COO)_3_· H_2_O Zn(CH_3_COO)_2_	Eu-ZnO	*P* = 15 MPa; *T* = 40 °C	15 nm	EB degradation	3.22 eV	Superior catalytic performance of SAS-prepared catalysts compared to those of commercial/traditional catalysts; complete discoloration after 240 min	[46]

**Table 3:** Synthesis of photocatalysts by sol–gel reaction and supercritical drying. SSA = specific surface area; RFCA = resorcinol-formaldehyde carbon aerogel; MB = methylene blue dye; MO = methyl orange dye; NPs= nanoparticles; RF= resorcinol-formaldehyde; SA: salicylic acid; s-PS = syndiotactic polystyrene; VOCs = volatile organic compounds; *P* = operating pressure; *T* = operating temperature; *t*= drying time.

Photocatalyst	Drying Conditions	Photocatalytic Application	Band Gap (eV)	SSA (m^2^/g)	Results	Ref.
TiO_2_ aerogel	-	SA degradation	-	600	About 98% of SA degradation in 900 min	[51]
TiO_2_ aerogel	*P* = 8 MPa; *T* = 40 °C; *t* = 6 h	Phenol degradation	3.03	464 ^a^; 100 ^b^	Optimum calcination temperature of 650 °C for the best photocatalytic performance (92% degradation yield); superior performance of scCO_2_- prepared samples than that of commercial TiO_2_	[80]
TiO_2_ aerogel	*P* = 8 MPa; *T* = 37 °C; *t* = 4 h	Methanol assisted water splitting	3.25	600 ^a^; 97 ^b^	Higher activity of scCO_2_- prepared samples than that of commercial TiO_2_	[79]
TiO_2_ aerogel	*P* = 10 MPa; *T* = 50 °C	Phenol degradation	-	112 ^b^	scCO_2_- prepared aerogel 3 times more active than commercial TiO_2_	[81]
TiO_2_ aerogel	*P* = 8.27 MPa; *T* = 35 °C	Water splitting	-	84.5 ^b^	H_2_ evolution rate 9.6 times higher with scCO_2_- prepared aerogel compared to that of commercial TiO_2_	[82]
TiO_2_ nanowires	*P* = 41.4 MPa; *T* = 60 °C; *t* = 24 h	MB degradation	2.5	-	MB degradation efficiency equal to 88%; higher activity of scCO_2_- prepared samples than that of commercial one	[84]
TiO_2_ layer	*P* = 14 MPa; *T* = 100 °C; *t* = 2 h	Stearic acid methyl ester decomposition	-	220 ^a^	Complete decomposition in about 22.5 h	[85]
TiO_2_ pillared clays	*P* = 20 MPa; *T* = 50 °C; *t* = 3 h	Phenol degradation	-	254 ^a^	Total degradation in less than 125 min	[86]
N-doped TiO_2_ aerogel	*P* = 20 MPa; *T* = 40 °C; *t* = 4 h	Phenol degradation	2.5	280 ^a^	45% of degradation after 180 min	[89]
Fe(III)-doped TiO_2_ aerogel	-	SA degradation	-	151 ^b^	TiO_2_- based aerogel with 1.8 at.% Fe(III) showed an apparent rate constant of SA degradation 6 times higher than commercial TiO_2_	[90]
TiO_2_-Pt aerogel	*P* = 9.5 MPa; *T* = 40 °C	Ethanol reforming	2.91-3.14	600 ^a^; 162 ^b^	The highest H_2_ production rate (7.2 mmol_H2_ h^-1^ g^-1^) with 1%Pt and the smallest particles size	[88]
TiO_2_–SiO_2_ aerogel	-	Water splitting	3.42	715 ^a^	H_2_ production in the range 0.73-1.35 mmol/g_TiO2_	[50]
TiO_2_–SiO_2_ aerogel	*P* = 16 MPa; *T* = 80-280 °C; *t* = 3 h	Benzene decomposition	-	967 ^a^	Benzene conversion up to 90% after 30 min	[92]
TiO_2_–SiO_2_ aerogel	*P* = 8.6 MPa; *T* = 50 °C	VOCs oxidation	-	469 ^a^; 306 ^b^	TiO_2_-SiO_2_ aerogel prepared by ethanol supercritical drying showed the highest removal efficiency (about 10%), also compared to commercial TiO_2_	[93]
TiO_2_–SiO_2_ aerogel	*T* = 50 °C	Trichloroethylene Oxidation		469 ^a^; 306 ^b^	TiO_2_-SiO_2_ aerogel prepared by ethanol supercritical drying showed the highest conversion (around 30%), also compared to that of commercial TiO_2_	[94]
TiO_2_ aerogel; TiO_2_–SiO_2_ aerogel	*P* = 11 MPa; *T* = 60 °C	Degradation of methanol	-	150; 635	98% conversion, almost double compared to commercial TiO_2_	[83]
TiO_2_–SiO_2_ pillared clays	*P* = 20 MPa; *T* = 50 °C; *t* = 3 h	Phenol degradation	-	400 ^a^	Lower activity of TiO_2_–SiO_2_ pillared clays compared to that of TiO_2_ pillared clays	[86]
TiO_2_–RFCA	-	MO degradation	-	645	More effective photocatalytic activity in the case of the TiO_2_–RFCA composites compared to the single materials	[95]
TiO_2_–WO_3_-Fe^3+^ aerogel	*P* = 11 MPa; *T* = 42 °C; *t* = 8 h	MB degradation	2.06	379 ^a^; 154 ^b^	About 90% and 70% of MB degradation after 12 h under UV or visible light, respectively	[49]
ZnO/s-PS	*P* = 20 MPa; *T* = 40 °C; *t* = 4 h	Phenol degradation	-	276 ^a^	Phenol removal increased by increasing the pH of the solution; synergy between photocatalyst and PS-based support assured robustness, chemical stability, easy recovery after treatment, high removal efficiency and selectivity	[96]
Au/TiO_2_ alginate beads	*-*	Water splitting	-	485–275 ^a^; 187–136 ^b^	Au/TiO_2_ alginate beads are 8 times more active under solar light than commercial TiO_2_ with the same Au amount	[87]
Au/CeO_2_ NPs dispersed on alginate aerogel	*-*	Water splitting	-	102	Photocatalytic activity of Au/CeO_2_ NPs under visible light outperform that of standard WO_3_ even under UV irradiation.	[31]
Ti-organic aerogel	-	Degradation of various dyes	3.4-3.2	688–350 ^b^	Effective stimuli-response of Ti-oxo-based materials	[97]
N-doped ZrO_2_/TiO_2_	-	MB degradation	-	56-94	N-doped samples show higher activity than undoped samples and commercial TiO_2_; the activity enhancement is higher in TiO_2_-based samples than Zr-modified ones	[91]
TiO_2_/RF polymer aerogel; TiO_2_/RF carbon aerogel	-	MO degradation	-	232–870	TiO_2_/RF carbon aerogel more active than TiO_2_/RF polymer aerogel in MO photodegradation	[95]

^a^ SSA evaluated before calcination; ^b^ SSA evaluated after calcination.

**Table 4:** Other applications of scCO_2_ in photocatalysis. AO7= Acid Orange 7; BY28: Basic Yellow 28; CNT= carbon nanotubes; MO = methyl orange dye; MOFs= metal−organic frameworks; MB = methylene blue dye; RGO= reduced graphene oxide; SSA = specific surface area of the photocatalysts.

Photocatalyst	scCO_2_ Use	Photocatalytic Application	SSA (m^2^/g)	Results	Ref.
TiO_2_-coated carbon surface	Supercritical pre-treatment before sol–gel reaction	MB degradation	378–487	Optimal conditions for the fastest MB degradation rate: MB concentration of 20 mg/l at pH 6, catalyst content of 2.5 g/l	[98]
TiO_2_-coated carbon surface	Supercritical pre-treatment before sol–gel reaction	Degradation of acid yellow	325–575	The degradation rate follows a pseudo-first order kinetics with the acid yellow concentration; it is proportional to the square root of the light intensity	[99]
TiO_2_-coated zeolite surface	Supercritical pre-treatment before sol–gel reaction	Degradation of Rhodamine B	103–267	Optimal conditions for the fastest degradation rate: Rhodamine concentration of 2 mg/l at pH 10, catalyst content of 6 g/l	[100]
TiO_2_-film supported on silicon substrate	Supercritical post-treatment	Decomposition of stearic acid	-	High photocatalytic efficiency with scCO_2_-treated films (complete decomposition after 75 min)	[101]
Ru-coordinated MOFs	Modification of MOFs structure	Hydrogen production	996–1257	scCO_2_-modified MOFs exhibited a higher activity than those of the pure MOF and MOF loaded with Ru particles	[102]
TiO_2_-loaded PCL foams	PCL foaming	Removal of textile dyes	-	Complete discoloration of AO7 and BY28 after 300 and 180 min, respectively	[103]
Au/ZnO layered structure on silk textile	Electroless plating and cathodic deposition of metal oxides on silk	Flexible wearable device	-	Issues related to conventional electroless plating overcome by sc-CO_2_: the silk was catalyzed without defects; adhesive property between silk and metalized-layer was enhanced	[109]
Ag/WO_3_ nanosheets	Supercritical exfoliation of WS_2_ nanosheets	Oxygen evolution reactions	-	Synergistic photocatalysis effect of Ag and amorphous WO_3_	[110]
Pd-doped TiO_2_	scCO_2_ as reaction medium for thermal hydrolysis	Reduction of CO_2_ to hydrocarbons	71–146	Pd-TiO_2_ exhibited CH_4_ and CO production rates up to 22 and 2 times higher than those of commercial TiO_2_	[104]
TiO_2_ powders	scCO_2_ as reaction medium for catalyst synthesis	Reduction of CO_2_ to formic acid	-	Optimal irradiation time of 5 h for the maximum yield of formic acid; the amount of formic acid increased with pH, up to almost 15 µmol/g_TiO2_ in a solution with phosphoric acid (almost pH 2)	[47]
CNT/TiO_2_ and CNT/ Cu-dopedTiO_2_	scCO_2_ as reaction medium for thermal hydrolysis	Reduction of CO_2_ to hydrocarbons	150–216	CNT/TiO_2_ composites showed CO and CH_4_ production rates (8.1 and 1.1 _µmol g^−1^ h^−1^, respectively) 4 and 15 times higher than those of commercial TiO_2_	[106]
TiO_2_ powders	scCO_2_ as reaction medium for thermal hydrolysis	Reduction of CO_2_ to hydrocarbons	40–152	TiO_2_ synthesis in supercritical medium resulted in a significant enhancement in the rate of CO_2_ catalytic conversion	[107]
TiO_2_ powders	scCO_2_ as reaction medium for thermal hydrolysis	MO oxidation	113–350	No effect of synthesis pressure on the activity of prepared TiO_2_; the increase of synthesis temperature from 200 °C to 300 °C led to an increase of crystalline quality and size, resulting in a higher activity	[105]
RGO/ZnO	Supercritical coating of RGO with ZnO, involving simultaneously “thermal decomposition of Zn(NO_3_)_2_ ·6H_2_O (i.e., ZnO precursor) + GO thermal reduction”	Hydrogen production	-	ZnO/RGO composite exhibited a H_2_ production activity 4.5 times higher (289 µmol/g) than that of pure ZnO (61.5 µmol/g) in 2 h	[111]
TiO_2_ powders	scCO_2_ as reaction medium for octanol oxidation	Octanol oxidation	50	Possibility to improve yield and selectivity by modulating temperature or pressure; above the critical point, photooxidative degradation rate increased as scCO_2_ pressure decreased at 36°C	[48]

**Table 5:** A summary about the applications and the advantages of using scCO_2_ in photocatalysis.

Applications	PROS
As a solvent for the deposition of semiconductors/active species on/in the support surface	Reduction or often complete elimination of the use of toxic organic solvents Preserving of porous structure of the supports without collapse High mass transfer Good and homogeneous dispersion of the metal-based compounds
As a solvent/antisolvent/co-solute in the production of particles-based powders	Reduction/elimination of the use of toxic organic solvents Good control of particles’ morphology and size by modulating the operating conditions Possibility to obtain very small size due to gas-like and liquid-like properties of scCO_2_ Very high surface area Total removal of organic solvents from powder
Production of aerogels	Total removal of organic solvents Porous structure is preserved, without collapse Very high surface area
As a foaming agent	No toxic organic solvents are used Possibility to control pores’ size by modulating the operating conditions Very high surface area Possibility to perform a one-step process “support foaming + deposition of organometallic precursors”
As a reaction medium	scCO_2_ is not flammable scCO_2_ is easy to recycle on an industrial scale scCO_2_ is less polluting than other solvents/reaction media rapid and complete removal of scCO_2_ by depressurization, avoiding the contamination of products Improvement in mass transfer Possibility to optimize the efficiency/selectivity by tuning the operating conditions and, consequently, the scCO_2_ properties Possibility to simultaneously perform also a supercritical coating/deposition, foaming, etc.

## Data Availability

Not applicable.
